# Heart rate responses, agreement and accuracy among persons with severe disabilities participating in the indirect movement program: Team Twin—an observational study

**DOI:** 10.3389/fspor.2023.1213655

**Published:** 2023-10-24

**Authors:** Andreas Jørgensen, Mette Toftager, Martin Eghøj, Mathias Ried-Larsen, Christina Bjørk Petersen

**Affiliations:** ^1^ National Institute of Public Health, University of Southern Denmark, Copenhagen, Denmark; ^2^ Department of Sports Science and Clinical Biomechanics, University of Southern Denmark, Odense, Denmark; ^3^ Centre for Physical Activity Research, Copenhagen University Hospital—Rigshospitalet, Copenhagen, Denmark

**Keywords:** adapted physical activity (APA), wearable technology, Team Twin co-running, heart rate (BPM), people living with disabilities, sensor accuracy, heart rate reserve

## Abstract

**Introduction:**

Heart rate (HR) monitors are rarely used by people living with disabilities (PLWD), and their accuracy is undocumented. Thus, this study aims to describe the HR response during the Team Twin co-running program and, secondly, to assess the agreement and accuracy of using HR monitors among PLWD.

**Methods:**

This 16-week single-arm observational study included 18 people with various disabilities. During the study, the subjects wore a Garmin Vivosmart 4 watch (wrist). To evaluate the agreement and accuracy we applied Garmin’s HRM-DUAL™ chest-worn HR monitors for comparison with the Vivosmart 4. The HR response analysis was performed descriptively and with a mixed regression model. The HR agreement and accuracy procedure was conducted on a subsample of five subjects and analyzed using Lin’s concordance analysis, Bland and Altman’s limits of agreement, and Cohen’s kappa analysis of intensity zone agreement. This study was prospectively registered at Clinical Trials.gov (NCT04536779).

**Results:**

The subjects had a mean age of 35 (±12.6), 61% were male, 72% had cerebral palsy were 85% had GMFCS V-IV. HR was monitored for 202:10:33 (HH:MM:SS), with a mean HR of 90 ± 17 bpm during training and race. A total of 19% of the time was spent in intensity zones between light and moderate (30%–59% HR reserve) and 1% in vigorous (60%–84% HR reserve). The remaining 80% were in the very light intensity zone (<29% HR reserve). HR was highest at the start of race and training and steadily decreased. Inter-rater agreement was high (k = 0.75), limits of agreement were between −16 and 13 bpm, and accuracy was acceptable (Rc = 0.86).

**Conclusion:**

Disability type, individual, and contextual factors will likely affect HR responses and the agreement and accuracy for PLWD. The Vivosmart 4, while overall accurate, had low precision due to high variability in the estimation. These findings implicate the methodical and practical difficulties of utilizing HR monitors to measure HR and thus physical activity in adapted sports activities for severely disabled individuals.

## 1. Introduction

People living with disabilities (PLWD), are heavily affected by their disability, which, for the majority, result in significantly poorer health, sedentary behavior, and premature death (compared to the general population). Premature death increases with severity of disability and with time (1–6). Adapted sports and physical activity (PA) have well-documented health benefits for PLWD (7, 8). Due to the functional limitations posed by disability, PLWD are often unable to participate in PA (9–14). Wheelchair-dependent individuals see the fewest opportunities for participation in PA (15). In Denmark, the Team Twin organization offers participation in *indirect* PA (*“an activity where a person unable to partake in physical activity on their own, is joined by someone who is able, and together partake in PA*” (16 p. 2)) for wheelchair-dependent persons, conducted in a social community consisting of PLWD and able-bodied volunteers. A previous pilot study from 2018 indicates that Team Twin participation is associated with increased social (e.g., connections and relations with peers and volunteers) and mental health (e.g., developing an identity) for PLWD subjects (17). The researchers monitored participants’ heart rate (HR) during Team Twin training sessions, further indicating intensity levels consistent with moderate to vigorous PA (17). Other indirect PA studies examine HR responses in therapeutic riding and power wheelchair soccer among PLWD, found similar HR-responses (18–20). Thus, there is an unresolved potential for severely disabled persons to gain physiological benefits from indirect PA. However, the evidence base is yet uncertain and more studies using rigorous and valid methodologies are needed to determine whether a physiological response occurs when participating in indirect PA.

Monitoring of HR (bpm), usually placed on the wrist or chest, is a non-invasive and commonly used proxy method for examining exercise intensity, and used in general populations, rehabilitation, and disease prevention programs (21). HR sensors have lately gained popularity in disability research (22). They are used to determine PA and intensity levels (21, 23), and chest ECG HR monitors have shown superior accuracy over wrist worn PPG, compared to the golden standard, standard medical ECG HR monitors (24–30). Recent reviews, evaluation designs, and adaptation of sports and rehabilitation programs find variance in the devices’ abilities (including accuracy and agreement) to assess biomechanical, locomotion, and physiological parameters of PA among PLWD, however mainly among high-performance athletes (e.g., Paralympics) (22, 31). Furthermore, the authors of the reviews address the major challenge of applying wearable technologies for PLWD resulting in a sizeable inter-subject variability and sufficiently lack of accuracy (22, 31). Moreover, the literature about HR monitors among severely disabled persons who participate in recreational PA is insufficient, and the validity of HR monitors could be challenged more due to various disabilities (affecting limbs, bodily posture, and movement). Lack of accuracy affected by time variations, wearing of the device, exercise type, context (laboratory vs. real world setting), individual behaviors, health condition, and applied devices (27–29, 32–35), highlights the importance of future research validating wearable sensors. Especially in specific populations, including PLWD (22, 27, 36), to assess the method suitability when using HR monitors to prescribe PA and intensity among PLWD who participate in recreation physical activities, where HR monitors are scarcely used.

Thus, this 16-week single-arm observational study primarily aims to describe HR response during participation of PLWD in the Team Twin co-running program and, secondly, to assess the agreement and accuracy of using HR monitoring in a subset of the population and context.

## 2. Materials and methods

### 2.1. Study design

This is an observational, single-arm study with no control group. The study is a sub-project from the “When Movement Moves”, described elsewhere (16). During a 16-week program, subjects wore a wrist-based HR monitor. The study was conducted in concordance with the Declaration of Helsinki (37), registered and approved by the Ethics Committee of Denmark’s Capital Region (Journal no.: H-20010668), including a pre-registration at Clinical Trials.gov (NCT04536779). All subjects gave written informed consent prior to inclusion.

### 2.2. Setting—Team Twin

The Team Twin organization provides an adapted running activity for PLWD (henceforth referred to as *handiathletes*) and has ten clubs across Denmark. It has approximately 450 members, 150 handiathletes and 300 volunteer-r*unners* (38).

The activity has the same format across the individual Team Twin clubs. A standard session is comprised of three phases, the before, during, and after phases. Sessions are typically scheduled on Sunday mornings. *Before*; runners and athletes meet and handiathletes are assisted in transferring from their wheelchair to running chair. The route is planned, and members and associates have casual conversations. *During;* co-running, handiathletes are sat in their running chairs driven by a runner. *After;* casual conversation, eating, and re-hydrating before end of session. The organization furthermore participate in numerous official races (half- and full marathons and other running events) national and international during their season.

### 2.3. Sample

We invited all 150 handiathlete members to participate. Between January and March 2021, 22 were enrolled (15%), representing six clubs. One withdrew after enrolment, and three either dropped out and or were excluded for non-adherence. Thus, 18 handiathletes were included in the analyses of this study (flowchart in Supplementary Figure S1). Inclusion criteria were 1) affiliation to a Team Twin club and 2) 18 + years of age. Characteristics of the sample were obtained between April and June, 2021. The study period began mid-June and finished in October 2021.

### 2.4. Wearables

All handiathletes were equipped with a Garmin Vivosmart 4 watch (39). The Vivosmart 4 was selected based on a practical approach taking the population into account. Following practical criteria was essential for the device selection, 1) suitable for relatively small wrists (due to disabilities) to fit all subjects properly, 2) comfortable to wear day and night for four months, to strengthen compliance, 3) long battery life (7 days) to avoid recharging daily and limit interference with the subject’s everyday life. The Vivosmart 4 did comprise the required features for the overall study (16) (sleep and HR monitoring) and the price accommodated the project budget. We personalized the devices based on the participants’ age, sex, height, and weight. All widgets (e.g., total steps per day and body battery (39)) were pre-emptively removed from the display to prevent interference. All obtained data were uploaded to individual Garmin profiles only accessible for the research team. All handiathletes, relatives and caretakers received a comprehensive walkthrough of the Vivosmart 4 (39). We employed the “Running” activity profile provided by Garmin (39) for tracking Team Twin sessions. We instructed the handiathletes and volunteer-r*unners* to activate/deactivate to track only during the actual co-running (the “during”-phase).

### 2.5. Heart rate measures

The Vivosmart 4 was placed on the athlete’s preferred wrist. It was worn both day and night, the latter to estimate resting heart rate (RHR) with the highest accuracy (40). RHR was estimated from the lowest 30-minute average calculated over the 24 h (40). We excluded the first two weeks of the RHR-data to gain further accuracy to accommodate a subject-specific calibration period (41).

We estimated peak heart rate (HR_max_) using Fernhall et al.’s formula (42), which takes disability into account. We calculated the individual heart rate reserve (HRR) by estimating a mean RHR across the whole period (excluding the calibration weeks described above) and subtracting it from the estimated HR_max_ (HRR = HR_max_-RHR). Based on the HRR, we determined %HRR (resulting in intensity levels) by the equation from Karvonen (43). Intensity levels based on HRR instead of HR-dependent estimation (e.g., %HR_max_) is recommended for avoiding over- or underestimation. The HRR method consider 1) *individual assessment*; which reflects the RHR, which differs from person to person, and thus less sensitive to over- or underestimation, 2) *accuracy in submaximal exercise*; more accurate to predict exercise intensity at submaximal intensity levels due to various factors like stress, fatigue, or medication use, and 3) *cardiorespiratory level*: individuals with higher fitness levels tend to have lower RHR resulting in a larger HRR and potentially higher exercise intensities. Thus, it is more appropriate for people with different levels of cardiovascular fitness to apply HRR estimation (21, 44).

To target individual HR intensity zones, we applied the intensity division from two guidelines (21, 45), and estimated HR for each participant reflecting the distribution from Table 1 (individual HR reflecting the intensity levels from Table 1 can be seen in Supplementary Table S1). We applied the predefined intensity zones and used the HR_max_ estimation from Fernhall et al. (42) as a frame to categorize HR responses in relation to the intensity of PA.

**Table 1:** The aerobic physical fitness classification applied in the study.

Intensity zones	%Heart Rate Reserve	Example activity^a^
Very light	<29	Sedentary
Light	30%–39%	Walking
Moderate	40%–59%	Brisk walking
Vigorous	60%–84%	Jogging
Very hard	>85%	Running fast/Maximum sprint

Intensity zone distribution (21, 45).

^a^ According to the general population.

### 2.6. Evaluation of agreement and accuracy

A convenience subsample of 5 subjects (*n* = 5) were recruited for the evaluation of device agreement and accuracy. The sample consisted of subject who voluntary agreed, were accessible, and able to wear the HRM-DUAL™. We compared the wrist-worn photoplethysmography-based Vivosmart 4 with a Garmin HRM-DUAL™ chest electrocardiogram-based HR monitor, connected to a Garmin *Forerunner 265*. Both devices were worn simultaneously during a Team Twin session, the *Forerunner 265* was placed on the running chair, not the athlete. Each athlete wore two monitors and acted as their own controls (concurrent validity). A researcher attached the wrist-worn and the chest-worn monitor as instructed (39, 46) and simultaneously activated/deactivated the monitors. During this study, we assessed the two devices using methods accommodating accuracy, agreement, and inter-rater agreement between the HR monitors (as described under 2.7.2. HR agreement—secondary objective).

### 2.7. Statistical approach

When using commercial HR monitors, including the one used in this study, continuous measurements (every second or other predefined time-interval) were not a standard output (24). The Vivosmart 4 sampling frequency is HR-determined instead of a pre-defined time interval. Meaning that the time frequency keeps in the same timeslot until the HR exceeds ±3 bpm. Thus, we used an HTML script to transform the HR data into a second-by-second time-interval dataset (see Supplementary File S1). Data was then trimmed with a ±15 s moving average filter, rounded to the nearest whole number, to accommodate interference, potential time displacement (when activating/deactivating the wrist/chest HR monitors), and device-specific measuring errors. This approach was applied to both datasets (wrist and chest). For the wrist data, we also compressed HR data into a one-minute mean and transferred the HR into %HRR (intensity levels) to fit the frame of intensity zones (Table 1). All statistical analyses were conducted with STATA version 17 (StataCorp LP, College Station, TX, USA). We applied a two-sided test with a significance level of 0.05% and 95% confidence intervals (16).

#### 2.7.1. HR responses—the primary objective

Descriptive statistics were used to determine time-in-zone (as described under 2.5—Heart rate measures). We performed a mixed regression analysis, with subjects as random effects, to investigate HR responses during and between training and races. Mean one-minute %HRR was the dependent variable, while time (in minutes), and race/training, serving as factor variables, were included with an interaction term being the independent variable in the model. It was adjusted for sex, age, and length of the individual training/race session and visually validated for Gaussian distribution, homoscedasticity, and best linear unbiased estimate of participant distribution. No violation of the assumptions was detected.

#### 2.7.2. HR agreement—secondary objective

We applied Lin et al.’s concordance correlation coefficient (Rc) to estimate accuracy and to detect deviance from the ideal trend line (the 45° concordance line) (47–49). Based on previous accuracy studies, we deemed a Rc > 0.80 acceptable to demonstrate accuracy between the wrist and the chest monitors (25, 26). Bland & Altman plots were used to estimate agreement between the two HR monitors within ±1.96 SD (50). Inter-rater agreement between the time spent at different intensity zones (Table 1) was evaluated with Cohen’s kappa coefficient (*κ*). We applied the coefficient thresholds from Landis et al. for the interpretation of the association, who characterized the values as <0 (no agreement), 0–0.20 (slight), 0.21–0.40 (fair), 0.41–0.60 (moderate), 0.61–0.80 (substantial), and 0.81–1 (almost perfect agreement) (51).

## 3. Results

### 3.1. Characteristics of the study population and program context

Our sample consisted of seven females and eleven males aged 19–65 (mean 35.4 years). In-depth characteristics are found in Table 2. Cerebral palsy (CP) was the most frequently self-reported diagnose (72%). Other conditions reported were muscular dystrophy, physical and mental disability, inherited neurodegenerative diseases, and multiple sclerosis (28%). Of those with CP, most were diagnosed with quadriplegia (92%), with a Gross Motor Function Classification System (GMFCS) at IV-V (85%). Due to their various conditions, eight handiathletes (44%) used both non- and prescription-only drugs on a daily base. These included, but were not limited to, Sirdalud, Euthyrox, Buscopan, medicinal cannabis etc. Common side-effects of these drugs might influence coronary circulation, resulting in palpitation, frequent pulse, increased RHR and extrasystole. During the 16-week program, the handiathletes had an average of seven sessions (±4) with the Vivosmart 4 activated and a mean HR of 90 (±16.8) bpm (20 ± 13.2%HRR) across race and training. An average session lasted 77 (±24) minutes and covered 10.5 (±4) km. The subsample (*n* = 5) used for the agreement evaluation consisted solely of males with GMFCS IV-V, had slightly higher mean HR during sessions, and a lower-than-average-sample BMI and weight (Supplementary Table S2).

**Table 2:** Characteristics of the study population and program context (*n* = 18).

Characteristics	Total sample (*n* = 18)
Age, (min, max)	35.4 ± 12.6 (19, 65)
Sex (male), *n* %	11 (61.1)
Disability, *n* %	
CP	13 (72.2)
Other	5 (27.8)
*(of those with CP) GMFCS*:	
III	2 (15.4)
IV-V	11 (84.6)
Medication with potential side-effects on HR, *n* %	
Yes	8 (44.4)
No	10 (55.6)
Body composition^a^	
Height (cm)	158.8 ± 10.6
Weight (kg)	57.7 ± 16.9
BMI (kg/m^2^)	22.8 ± 4.8
Heart rate data from Vivosmart 4	
RHR (bpm)^b^	69.9 ± 9.8
HRR (bpm)	105.8 ± 7.3
HR_max_ (bpm)^c^	175.5 ± 7.0
Team Twin sessions & context	
Training session participation	9 ± 5
Race session participation	2 ± 1
Sessions with an activated Vivosmart 4	7 ± 4
Mean HR during training/race (bpm)	90 ± 16.8
Mean %HRR during training/race	20 ± 13.2
Duration (min)	77 ± 24
Distance (km)	10.5 ± 4

Data are presented as mean ± SD, unless stated otherwise.

CP, cerebral palsy; GMFCS, gross motor function classification system; BMI, body mass index; HR, heart rate; RHR, resting heart rate; HRR, heart rate reserve; HRmax, heart rate max; %HRR, percent of heart rate reserve (intensity); bpm, beats per minute.

^a^ *n* = 16 due to missing data.

^b^ Mean from the first 7-day period after the two-week calibration.

^c^ Estimated from Fernhall et al. (42).

### 3.2. HR-responses

A total of 130 sessions, 113 training (162 h, 18 m, and 46 s), and 17 races (39 h, 51 m, 47 s), gave a total monitoring time of 202:10:33 (HH:MM:SS) recorded with the Vivosmart 4. Table 3 depicts time spent in each intensity zone (cf. Table 1). Most time (80%) was spent in the *very-light* intensity zone, which equals sedentary behavior. All subjects sustained an HR response consistent with *light* and *moderate* intensity zones; however, the duration in each level varied considerably across the group. Nine handiathletes (50%) reached the *vigorous* intensity zone at one or more points during the program period, and the duration in it varied between 0.02% and 6.6% of their total recorded time (Supplementary Figure S2).

**Table 3:** Total time and time proportions spent in each intensity zone during Team Twin activity measured by wrist HR monitor.

HR intensity zones	Very light	light	Moderate	Vigorous	Very hard	Total Time
HH:MM:SS	161:26:42	21:06:23	18:03:09	1:33:52	0:00:40	202:10:33
Proportion in each intensity zones	80%	10%	9%	1%	0%	100%

Intensity zone distribution as in Table 1 (21, 45).

**Figure 1:**
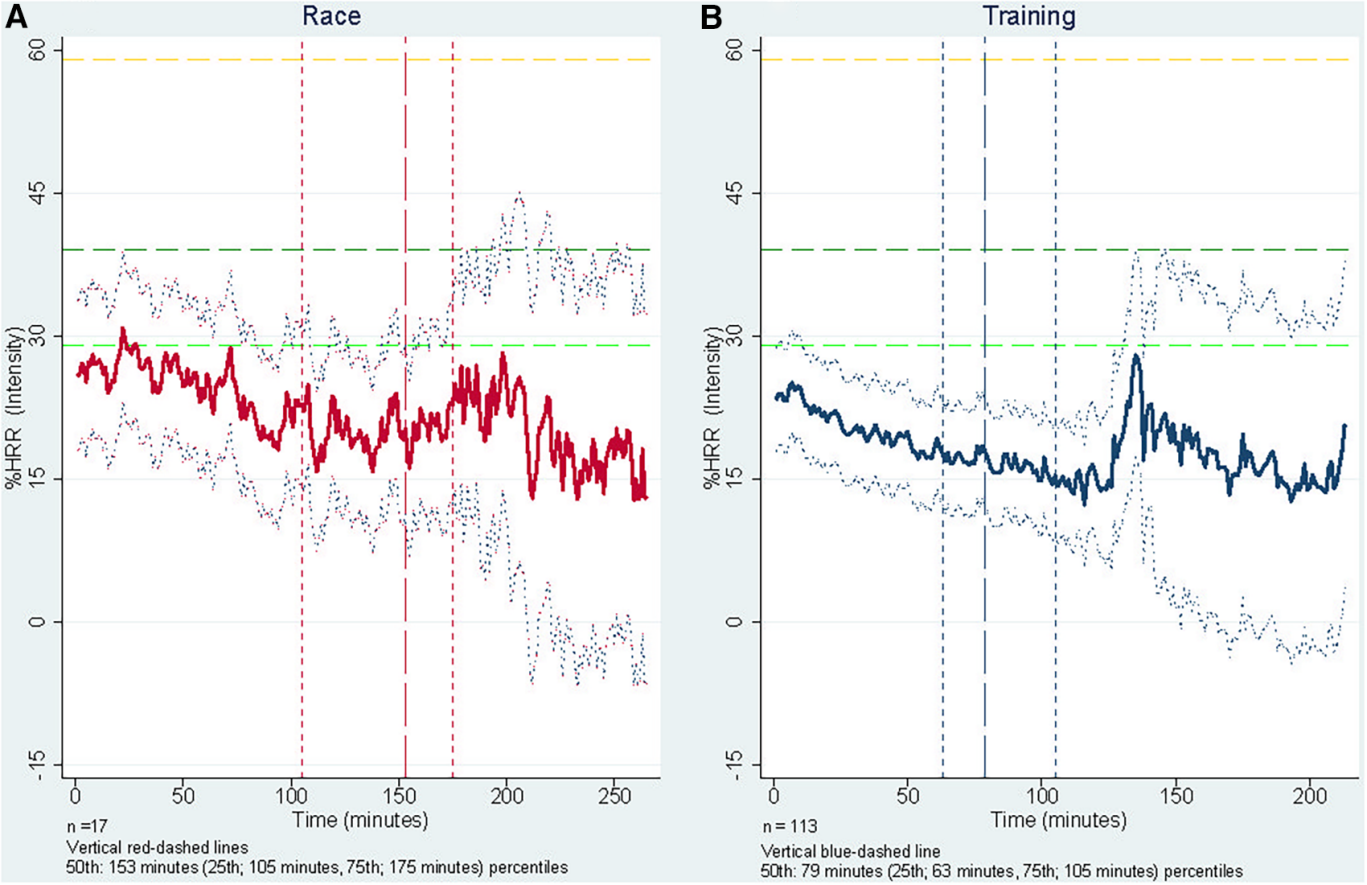
One-minute mean of %HRR-responses (intensity) during training (*n *=* *113) and races (*n *=* *17), adjusted for age, sex, and length of session. The figure illustrates %HRR-responses (intensity) for Race (**A**) and Training (**B**), combined for all handiathletes. The y-axis is %HRR and the x-axis is time in minutes. The yellow (top), dark green (middle) and light green (bottom) dashed vertical lines indicates the threshold for *very*-*light* (<29% HRR), *light* (30%-39% of HRR) and, *moderate* (40%–59% of HRR) intensity levels reflecting Table 1. The red- and blue-dashed vertical lines represents the x-axes 25th, 50th, and 75th percentiles, respectively.

We found a decline-in-HR-over-time tendency during both training and races. This tendency is depicted in Figure 1, which presents the %HRR-responses for all handiathletes grouped by race or training participation. Further, the plot illustrates a variance in the %HRR with increasing duration, meaning that dispersion increases with time. The median training duration was 79 min (25 p; 63 min, 75 p; 105 min), and the median race duration was 153 min (25 p; 105 min, 75 p; 175 min) (Figure 1). The greater dispersion after the 79th and 153rd min, respectively, are likely due to significantly fewer observations, as few handiathletes had training/race sessions lasting for more than 105 and 175 min (75% percentile), respectively. During race participation, we found a higher mean %HRR (intensity level), compared to training sessions, 26%HRR (95%CI 20.9; 31.0) vs. 19%HRR (95%CI 14.1; 24.0), respectively. A significantly higher mean of 6.8%HRR (95%CI: 6.1; 7.4, *p* < 0.000) adjusted for sex, age, and duration of the session, in favor of race participation was found (see graph in Supplementary Figure S3). A sensitivity analysis revealed the same results when only including handiathletes who had data from both race and training sessions (*n* = 13) (data not shown).

### 3.3. Agreement and accuracy between the wrist- and the chest-worn heart rate monitor used among PLWD

A total of 11:26:25 (HH:MM:SS) (equaling 41185 HR values) were collected for the second-by-second analysis, evaluating the agreement between the wrist and chest monitors. The mean time subjects wore the devices was 01:55:04 (min; 01:02:09, max; 02:37:38) (HH:MM:SS). We conducted six wrist and chest evaluations on five handiathletes. Two out of the six evaluations were conducted during a race.

**Figure 2:**
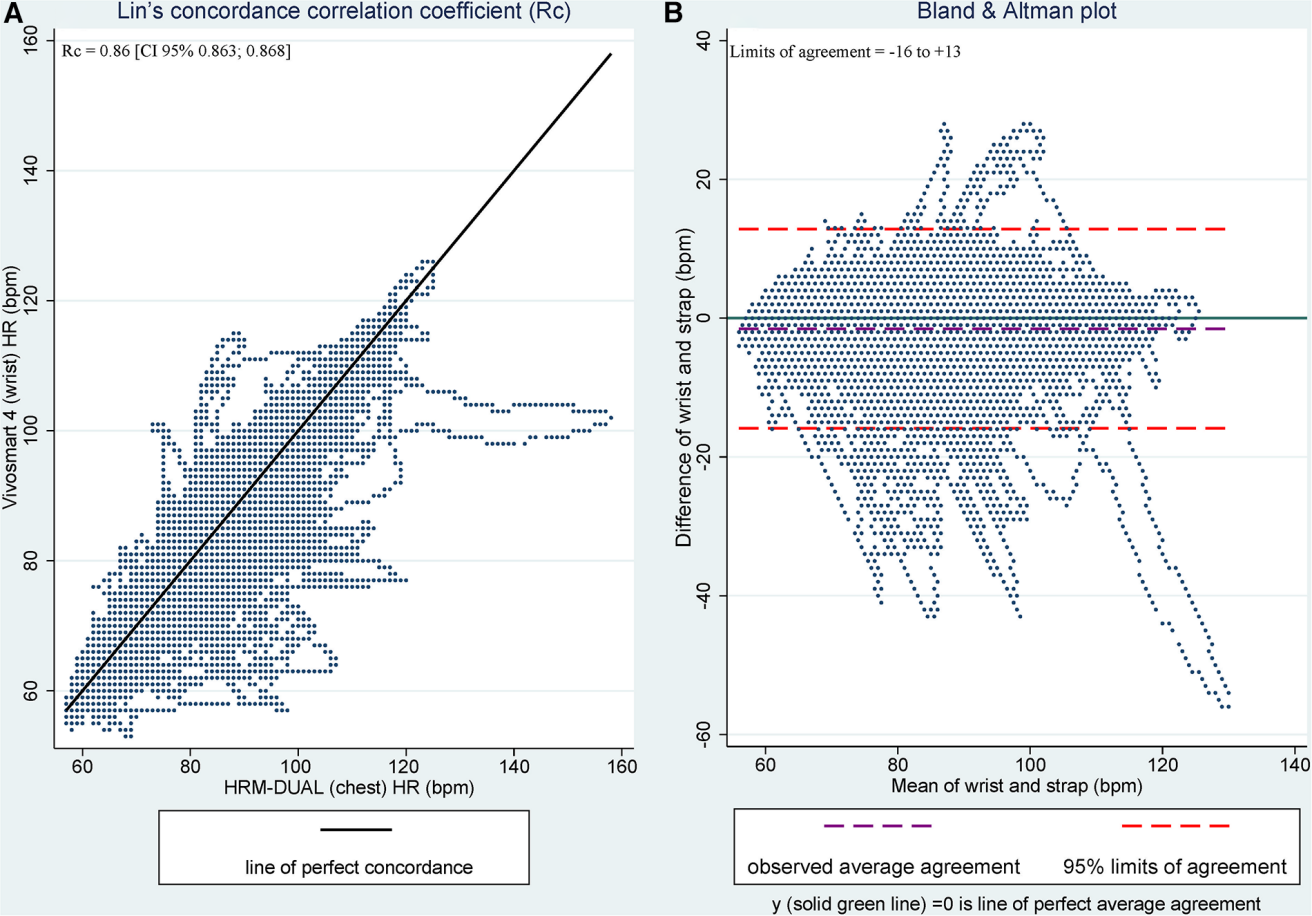
Lin’s concordance correlation coefficient (**A**) and Bland & Altman plot (**B**) for wrist and chest agreement and accuracy analysis. Figure 2A illustrates Lin’s et al. concordance correlation coefficient (Rc) data point arounds the 45° concordance line. Figure 2B represents the Bland & Altman plots, estimating total agreement within ±1.96 SD between the wrist and chest HR monitors (Vivosmart 4 and HRM-DUAL™, respectively).

Across the evaluation of agreement and second-by-second analysis, the wrist monitor showed an acceptable mean accuracy with the chest HR monitor [Rc = 0.86 (CI 95% 0.863; 0.868)] (Figure 2A). The Bland & Altman plot reveals that dispersion occurs during the whole spectrum of HR intensities; however, more excellent dispersion with increasing HR. That means, with an increase in HR, the agreement between the two monitors decreases, resulting in greater heteroscedasticity. The limits of agreement between the wrist and the chest monitor fall within −16 and +13 bpm. Despite an overall acceptable accuracy, the individual variation from Lin’s Rc-coefficient and the agreement from the Bland & Altman plots vary notably (Rc between 0.05–0.91 and Bland & Altman plot between −27 and +18, see Supplementary Figure S4). Cohens kappa’s coefficient reveals a *substantial* inter-rater agreement between the two HR monitors of the intensity zones (k = 0.75), with an overall agreement of 92%. The chest monitor generally registers more time at higher intensity zones than the wrist monitor (e.g., total time in *moderate*-intensity zone intercepted for the wrist HR monitor; 0:53:12 (HH:MM:SS), and for the chest HR monitor; 1:01:46 (HH:MM:SS)). The distribution and inter-rater agreement between the Vivosmart 4 and the HRM-DUAL™ are available in Supplementary Table S3.

## 4. Discussion

The primary findings include that PLWD elicit heart rate responses equal to *very light* intensity zones (sedentary) with intensity fragments in *light* to *moderate* zones and sporadically *vigorous* intensity, with a considerable variation in the duration of higher intensities across the sample when participating in the Team Twin co-running *indirect* activities. Moreover, evaluating the agreement and accuracy of the Vivosmart 4 we observed that the wrist-worn monitor provided an overall acceptable accuracy, agreement, and *substantial* inter-rater agreement. compared to the chest worn HRM-DUAL™; however, it was not consistently precise.

The Team Twin pilot study of 2018 (17) indicated that two out of three subjects reached HR levels consistent with the *moderate* intensity zones, sustained for long periods and sometimes *vigorous* intensity zone during training sessions and official races (17). It concluded that HR responses were consistent with a cardiorespiratory fitness impact (17) due to arousal and locomotion (isometric muscle stimulation). Our findings, however, contradict the notion, as only a limited amount of the total recorded time reached *moderate* intensity zones or above. We identified spikes in the HR of selected individuals, sustaining intensity levels above the *very*-*light* intensity zone (sedentary), but they were few. Reflecting on the Team Twin co-running activity (movement by sitting in a chair pushed by others), a significant proportion of HR correlating to sedentary behavior was expected. Although no other studies evaluating Team Twin were discovered, prior studies evaluating HR response for individuals with CP and quadriplegia participating in *indirect* adapted sports exist. A study on power wheelchair sports has shown HR levels of both *light* (20) and *moderate* intensity (19). One study indicated that the type of disability (e.g., cerebral palsy (including subtypes (52) and muscular dystrophy) influenced HR responses, where persons with cerebral palsy reached a higher HR response than people with other types of disabilities (19). Same pattern of increased intensity levels (%HRR) during therapeutic riding was observed among CP diagnosed persons and correlated with increased GMFCS-levels (18). Older studies in this field document significantly higher energy expenditure among persons with cerebral palsy when performing the same activities as able-bodied peers (53–56). Thus, the reason for some handiathletes reaching higher intensity levels may be attributed to a disability-specific influence.

It is worth noting, however, that as handiathletes are sedentary in the Team Twin program, the assumption that their HR response will correspond expectedly to that of physical activity, which is defined as “*any bodily movement produced by skeletal muscles that requires energy expenditure*” (7 p. 15), may not be just uncertain; it might be erroneous to use HR as a proxy for PA in this specific population and in this context.

An argument can, however, be made that the usual disability-dictated locomotive patterns (of CP) may be triggered to a greater extent during Team Twin sessions, as the running chair encounters obstacles, bumps, turns and moves with a higher pace than usual in their everyday life. This could, however speculative, result in hyperreflexia, increased muscle tone, isometric muscle stimulation, and involuntary and uncontrolled movement, as indicated in the pilot study (17) and discussed in the studies of power wheelchair activities (19, 20). The stimulation from the activity could, strictly speaking, affect their bodies and provoke higher energy expenditure, resulting in HR responses, despite participating in otherwise sedentary activity (19, 53). However, further investigation with different methods is needed to determine if a greater rate of energy expenditure is at play. Other explanations of the increased HR responses include disability type (19), medication use, psychological arousal (extra-sensory perception), the activation of the sympathetic nervous system from adrenaline rush due to emotions such as excitement, nervousness, fear, anxiety, fidgeting, or joy (33, 34, 44, 57). All factors are likely to impact HR response, combined with the well-known individual factors affecting cardiorespiratory fitness (age, genetic, sex, social, and psychological factors (21)), contributing to the HR response identified here. This explanation should be seen in the light of the steadily decreasing HR response from start to finish (Figure 1), where feelings like expectation, excitement, and nervousness at the beginning of a co-running session may be more strongly at play than the (physical) activity itself. The pilot study on Team Twin described the exaggerated and increased movement due to the excitement, joy, and happiness inherent to participation in the co-running sessions (17), which may partly explain the increase in HR. Our findings support the notion, as we observed significantly higher HR response during official races than in regular Team Twin training sessions, where the context is different, and stakes may be perceived as higher. During races, handiathletes might experience affection from cheering spectators, an excited atmosphere, and a competitive setting, increasing their excitement and, in turn, movements in the upper body and limbs. Thus, the combination of increased within-chair movement, psychological arousal, disability- and (potential) medication-induced HR responses, race/training context, and individual variation are likely to contribute to HR responses. Considering these factors and the sedentary nature of the Team Twin activity, the definition of PA (7) and the (declining) HR response (Figure 1) observed in this study further complicates the questions of whether this is energy expenditure (PA) or other factors-induced HR responses, more than the Team Twin co-running program activity itself or a combination of multiple factors.

### 4.1. Heart rate agreement and accuracy

Lin’s Rc coefficient revealed a mean acceptable accuracy between the wrist and the chest HR monitor across all subjects, consistent with similar studies (24, 25, 27). Our study, however, is the first evaluation of this specific wearable (Vivosmart 4) and population (24–30, 35). The limit of agreement falls within −16 and +13 and does not reveal any systematic bias or variability. Sartor et al. state that ±15 bpm is a great limit of agreement considering a second-by-second comparison (27) while the present study shows greater accuracy than other comparison studies (24, 25, 58, 59). HR accuracy studies among the general population imply that an increase in HR is possibly associated with increased upper-body movement (24, 32) and types of exercise involving more movement in the limbs (e.g., use or no use of arms on an elliptical trainer), highly affects accuracy among different wrist HR monitors (25, 26). However, some studies do not find the same inaccuracy-error regarding intensity and type of exercise (28, 29). These inconsistencies are likely due to methodology, protocol, and individual differences. In the present study, we observed a great individual variance in accuracy among the subjects included in the evaluation (see Supplementary Figure S4). This finding could be due to errors caused by movement, and in this case, by disability-induced upper-body (uncontrolled) movement and flexion of the wrist. The difference between the handiathletes with the most and least wrist/chest agreement was seen among handiathlete C and F (see Supplementary Figure S4). Their individual disability-specific conditions and diagnoses differed significantly. One lives with a disability prognosticate great hypokinesia (no muscle activity and thus no movement in limbs), and the other with an appearance similar with CP induced dyskinesia (characterized by rapid involuntary and abnormal movement (52)). Thus, it is likely that the disability-specific conditions affecting locomotion result in major subject-specific-agreement and variation (22), coupled with other factors affecting accuracy, as described by Garmin (e.g., non-proper fit on the wrist, physical characteristics and type of exercise, and intensity of the activity (60)). Although the chest HR monitor seems more sensitive to higher intensity levels compared to the Vivosmart 4, most of the time was captured within the same intensity zones (92% agreement). Further, the Vivosmart 4, used among PLWD, does not reach the same accuracy as other wrist-worn HR devices have demonstrated among the general population (e.g., Apple Watch and Mio Fuse (24–26, 29)). Thus, HR accuracy and agreement seems to be highly affected by individual variation and disability characteristics and can, for some individuals, cause measurement errors.

### 4.2. Methodological consideration and limitations

A main strength of this study is the second-by-second comparison of HR-data (27). Additionally, applying the HRR to estimate intensity levels, rather than a standard formula (e.g., % of HR_max_) strengthens the validity of each individual (21, 34). Applying the chest-worn monitor as the reference device seems reliable due to a high overall accuracy between chest monitors and golden standard electrocardiograms for HR monitoring (24–30, 35, 36), especially under the circumstances of “real world settings” where other HR validation studies also apply commercial chest worn HR monitors (61–63). However, a limitation of the study may be that no validation studies have examined the Garmin HRM-DUAL™ among the general population nor among PLWD.

The study also faced other limitations. We only managed to recruit five subjects and conducting a total of six evaluations due to limited resource and time and difficulties of recruiting the handiathletes. Some of the reasons for the sparse subsample (*n* = 5) was 1) few available days (only Sundays) due to not all planned visits were allocated to conduct the evaluation (some visits where scheduled for interviews and observation), 2) difficulties attaching the strap to all subjects due to their many layers of clothes and disabilities, and 3) some subjects refused to partake while the felt uncomfortable getting undressed to get the strap attached. Thus, our data basis is quite small and expected to be selection bias.

Despite applying the most valid methods available for the HRR estimation, a total of 7 h and 19 min was registered below the estimated RHR (<0%HRR) (data not shown). This is why we see 95% CI below zero in Figure 1. This questions 1) the accuracy of the estimated RHR from the Vivosmart 4, 2) the accuracy of the Vivosmart 4 during Team Twin activities, 3) the methods we applied to easily (and accurately) estimate HR responses among PLWD, and 4) the influence of the potentially falsely increased RHR due to the side-effects of drugs among 44% of the handiathletes (Table 2) and the well-known CP-induced increased RHR (64). During a *post hoc* estimation, we observed that handiathletes who used drugs with potential side-effects on the coronary circulation and pulse obtained significantly higher HR intensity zones than those who have not been prescribed those drugs (data not shown). This elaborates further the questions of HR as a measurement for PA-induced energy expenditure among PLWD with increased use of medications (with potential effects on HR).

A significant limitation of the study is that HR_max_ could not be directly measured, as no handiathletes reached peak HR. HR_max_ estimations are acceptable when no direct HR_max_ measure is feasible (21). Thus, the equation from Fernhall et al. (42) was used to estimate HR_max_ in relation to the selected intensity thresholds (21, 45). Using more wide-spread equations for HR_max_ estimation (e.g., Gellish et al. (65) or Fox et al. (66)), the proportion of time spent in higher intensity zones decreased (see Supplementary Table S4). However, apart from the v*ery hard* intensity zone, all intensity levels were generally alike, regardless of the method used to estimate HR_max_. In general, quantifying intensity zones and interpreting HR response as an assumed function of PA among this population, with the accessible tools, applied methods, and taken approaches, are highly challenging, however, we believe we have optimized the use of the chosen techniques and methods. Ultimately, the level of accuracy will depend on the specific use and activity performed by the user.

Subjects faced significant challenges in operating the Vivosmart 4 due to the incompatibility between its design (tiny and touch screen (39)) and disabilities impairing both fine and gross motor function. This meant that turning on/off the device during sessions relied on runners’ and relatives’ help. Still, remembering to activate the watch was challenging, and much data was not monitored compared to the actual participation rate (Table 2). A larger device with buttons instead of a touchscreen would likely have better accommodated the participants’ motor function. However, a wrist-worn wearable seems more suitable, compatible, and accessible for this population than a chest-worn monitor due to the challenge of attaching it on severely PLWD.

Some handiathletes did not wear the watch precisely as described in the manual (39). During observations at Team Twin training sessions, we noted an improper fit of the watch (too loose/tight and wrong position on the wrist (39)). Sometimes, the PPG lens was dirty—which could impact its accuracy. The placement on either the left or right wrist could also affect the accuracy, although prior studies on the general population did not find accuracy variation between the left and right wrist (25). However, Garmin highlights that “wrist flexing” can prohibit accuracy (60), which inhibits the agreement and accuracy Thus, in a population with hyperreflexia due to CP attaching the HR monitor to the less affected side should be considered.

Garmin also highlights some essential factors to accommodate optimal conditions for the chest-monitor-accuracy (e.g., being wet to maintain electrical connection, correct positioning, warming up, not wearing synthetic fabrics due to the development of static electricity etc (46)). Some of those issues were difficult to accommodate among the PLWD, e.g., optimal chest positioning, due to abnormal posture and contact between monitor strap and running chair-back changing its fit over a session.

The findings from this study are limited to the specific population and activity form of recreational indirect PA regarding the external validity. However, our reflections based on the use of HR-monitors among PLWD, and the methodological challenges can be generalized to other groups of PLWD, with specific conditions that could affect the validity of a HR monitor. Despite that, generalize conditions across various disabilities in relation to this specific topic may be challenging, due to all the different factors affecting HR monitoring. Thus, applying and selecting HR monitors (either wrist- or chest-worn) among population with specific conditions should be considered according to the issues addressed in the study.

## 5. Conclusions

People with severe functional limitations participating in the Team Twin co-running program demonstrate HR responses consistent with time spend mostly in *very-light* intensity. The Vivosmart 4 reached acceptable accuracy, but low precision as the estimate was highly variable. Our study further implicates major methodological challenges and suitability when using HR as a proxy for PA and HR monitors among severely disabled persons participating in indirect adapted sports activities. Future studies, practitioners, or health professional applying HR monitors among individuals living with severely disabilities, should consider how individual conditions may challenge the accuracy of the HR monitors and thus, the selection including attachment of the devices.

## Data Availability

The original contributions presented in the study are included in the article/**Supplementary Material**, further inquiries can be directed to the corresponding author.

## Supplementary material

Supplementary File S1, Supplementary Figure S1, Supplementary Figure S2, Supplementary Figure S3, Supplementary Figure S4, Supplementary Table S1, Supplementary Table S2, Supplementary Table S3, Supplementary Table S4 HR transformation example. Flowchart. Time spend in each intensity level per participant. Training vs. Race bar graph. Individual Bland & Altman plots. Intensity levels estimated for each participant. Subsample and remaining sample characteristics. Cross tabulation Cohen’s kappa coefficient. HRmax estimations influence on interpretation.
