# Supplementary material for: Heart rate responses, agreement and accuracy among persons with severe disabilities participating in the indirect movement program: Team Twin—an observational study
Source: Front Sports Act Living. 2023 Oct 24;5:1213655. doi: 10.3389/fspor.2023.1213655 (PMC10627970; doi:10.3389/fspor.2023.1213655)
Supplement: Supplementary File S1, Supplementary Figure S1, Supplementary Figure S2, Supplementary Figure S3, Supplementary Figure S4, Supplementary Table S1, Supplementary Table S2, Supplementary Table S3, Supplementary Table S4 — HR transformation example. Flowchart. Time spend in each intensity level per participant. Training vs. Race bar graph. Individual Bland & Altman plots. Intensity levels estimated for each participant. Subsample and remaining sample characteristics. Cross tabulation Cohen’s kappa coefficient. HRmax estimations influence on interpretation. [file Datasheet1.pdf]

Supplementary Material

Heart rate responses, agreement and accuracy among persons with severe disabilities participating in the indirect movement program: Team Twin - An observational study

Andreas Jørgensen*, Mette Toftager, Martin Eghøj, Mathias Ried-Larsen, and Christina Bjørk Petersen

*** Correspondence:**
Andreas Jørgensen
ajor@sdu.dk

1 Supplementary Data

None available

2 Supplementary Figures and Tables

Figure S1: title; Flowchart.
Figure S2: title; Time spend in each Intensity zone per participants.
Figure S3: title; Training vs Race bar graph.
Figure S4: title; Individual Bland & Altman plots.

Table S1: title; Intensity levels estimated for each participant.
Table S2: title; Subsample and remaining sample characteristics.
Table S3: title; Cross tabulation Cohen's kappa coefficient.
Table S4: title; HRmax estimations influence on interpretation.

File S1: title; HR transformation example.

2.1 Supplementary Figure 1. Flowchart of the included participants

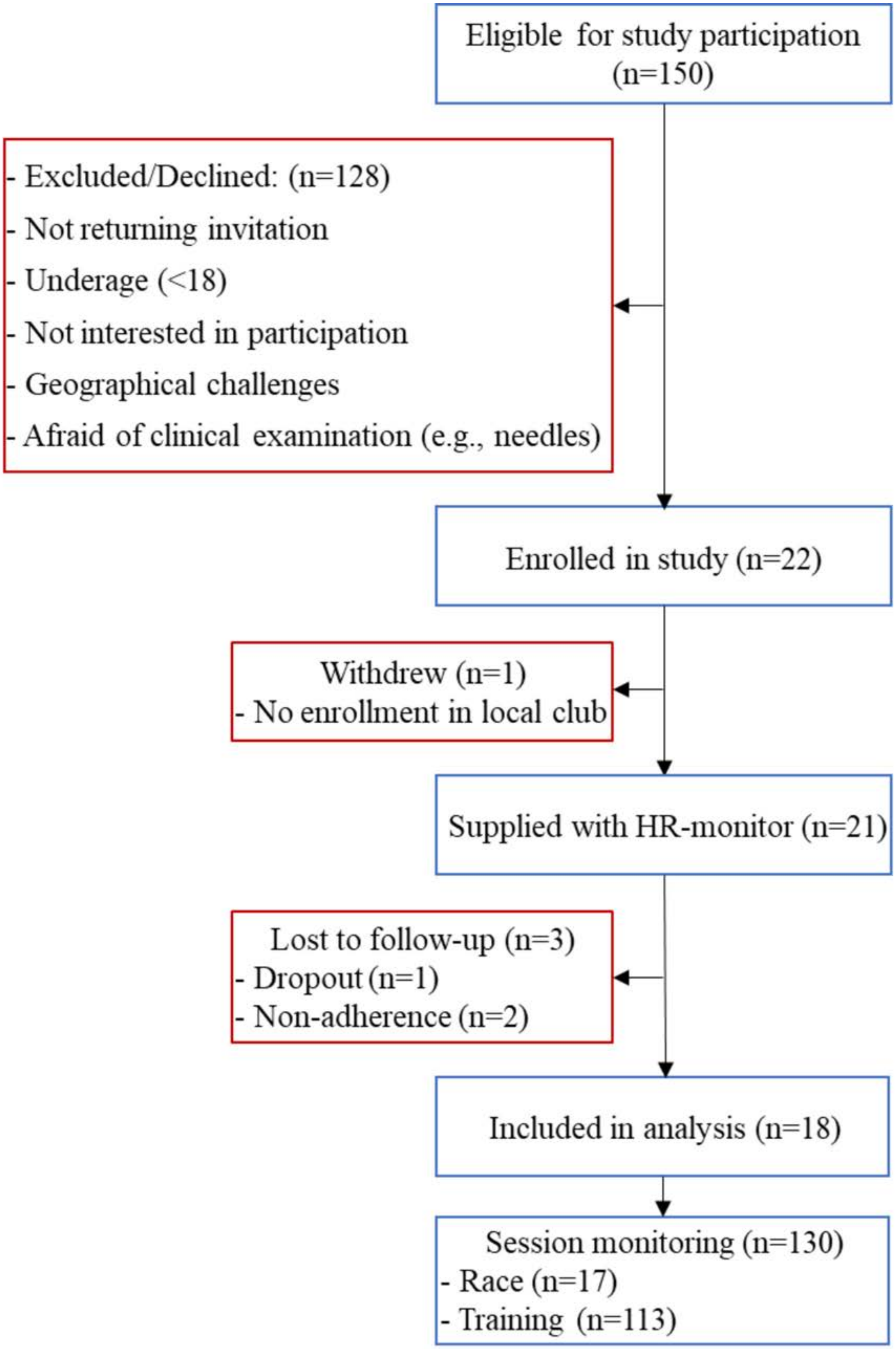

2.2 Supplementary Figure 2. Time spent in each intensity zone per participant

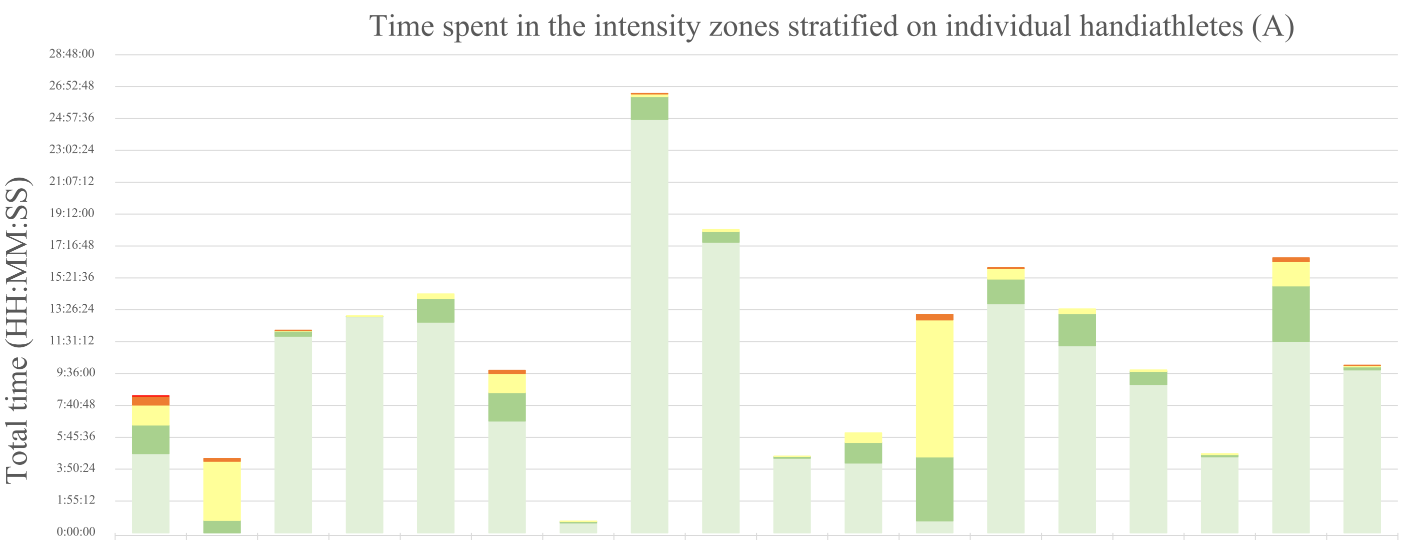

	ID01	ID02	ID03	ID04	ID05	ID06	ID07	ID08	ID09	ID10	ID11	ID12	ID13	ID14	ID15	ID16	ID17	ID18
Very hard	0:00:40	0:00:00	0:00:00	0:00:00	0:00:00	0:00:00	0:00:00	0:00:00	0:00:00	0:00:00	0:00:00	0:00:00	0:00:00	0:00:00	0:00:00	0:00:00	0:00:00	0:00:00
Vigorous	0:32:40	0:10:21	0:00:08	0:00:00	0:00:00	0:11:13	0:00:00	0:01:50	0:00:00	0:00:00	0:00:00	0:19:48	0:03:18	0:00:00	0:00:00	0:00:00	0:12:57	0:01:37
Moderate	1:12:21	3:33:44	0:02:43	0:01:00	0:16:29	1:09:13	0:02:06	0:09:36	0:07:40	0:00:21	0:34:27	8:15:34	0:37:54	0:17:38	0:04:47	0:03:45	1:28:39	0:05:12
Light	1:43:16	0:45:02	0:18:18	0:02:11	1:25:49	1:42:57	0:04:44	1:22:32	0:38:17	0:06:52	1:14:13	3:50:28	1:29:21	1:56:04	0:47:28	0:07:56	3:20:15	0:10:40
Very-Light	4:46:13	0:00:12	11:50:51	13:01:02	12:41:22	6:44:07	0:36:06	24:54:09	17:30:35	4:29:34	4:12:38	0:43:42	13:47:45	11:15:53	8:56:24	4:34:50	11:32:05	9:49:01

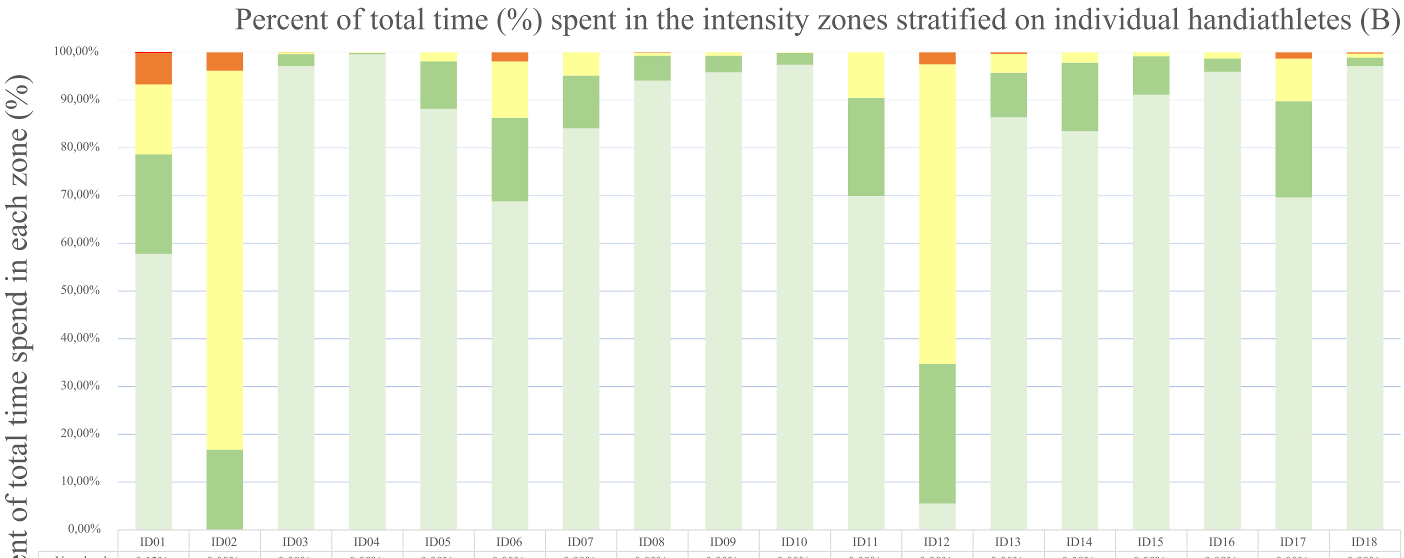

	ID01	ID02	ID03	ID04	ID05	ID06	ID07	ID08	ID09	ID10	ID11	ID12	ID13	ID14	ID15	ID16	ID17	ID18
Very hard	0,13%	0,00%	0,00%	0,00%	0,00%	0,00%	0,00%	0,00%	0,00%	0,00%	0,00%	0,00%	0,00%	0,00%	0,00%	0,00%	0,00%	0,00%
Vigorous	6,60%	3,84%	0,02%	0,00%	0,00%	1,91%	0,00%	0,12%	0,00%	0,00%	0,00%	2,51%	0,34%	0,00%	0,00%	0,00%	1,30%	0,27%
Moderate	14,61%	79,36%	0,37%	0,13%	1,91%	11,78%	4,89%	0,60%	0,70%	0,13%	9,54%	62,77%	3,95%	2,18%	0,81%	1,31%	8,92%	0,86%
Light	20,85%	16,72%	2,50%	0,28%	9,94%	17,52%	11,02%	5,20%	3,49%	2,48%	20,54%	29,19%	9,32%	14,34%	8,06%	2,77%	20,15%	1,76%
Very-Li..	57,80%	0,07%	97,11%	99,59%	88,16%	68,79%	84,08%	94,08%	95,81%	97,39%	69,92%	5,53%	86,38%	83,49%	91,12%	95,92%	69,63%	97,12%

Graphs (A) and (B) demonstrate how much time each handiathlete spent in each intensity zone during the 16 weeks of training and race sessions. Graph A (top) represents the actual amount of time spent. In graph B (bottom), time is expressed as a percentage of total time (%)

2.3 Supplementary Figure 3. Training vs Race bar graph

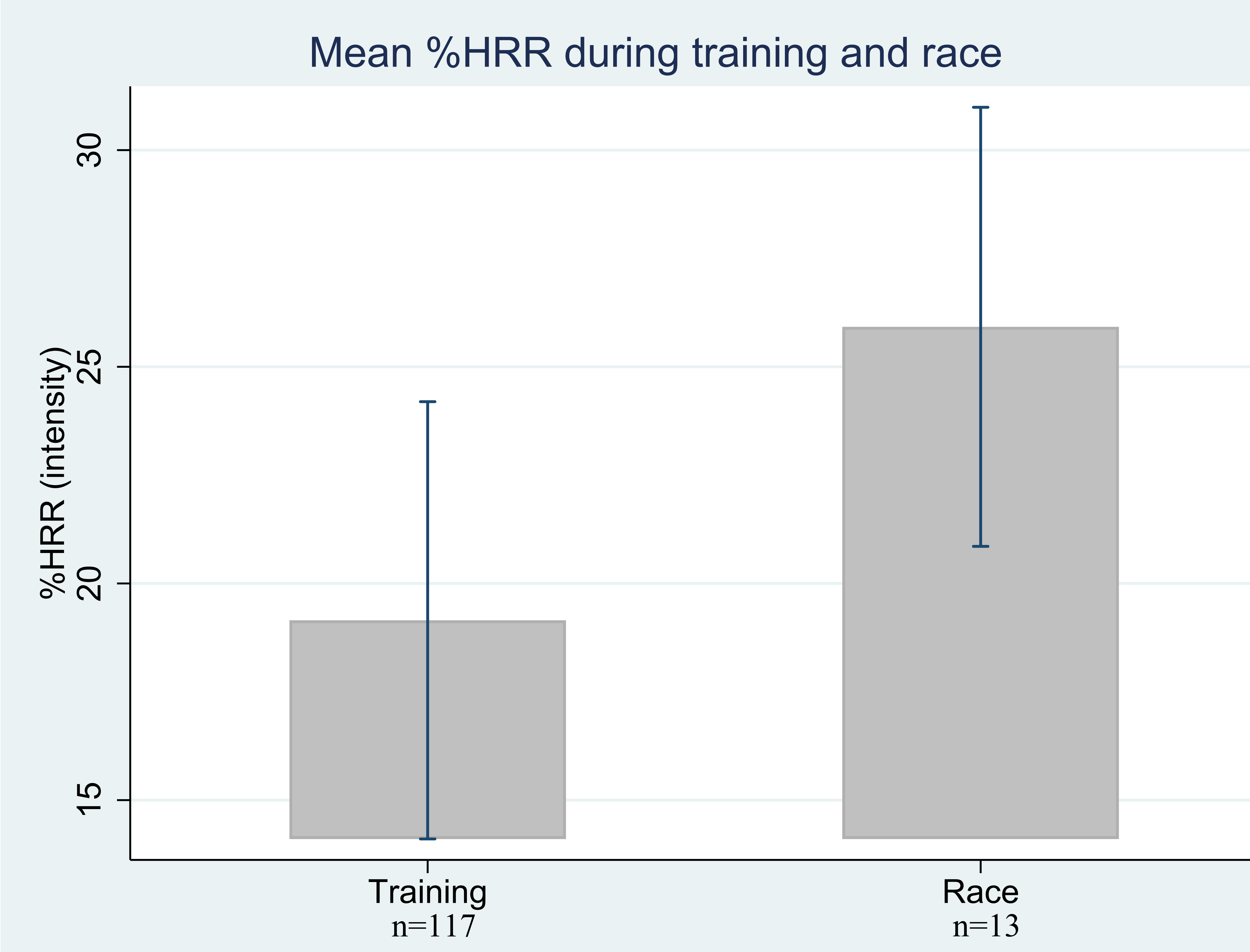
Mean %HRR during training and race
%HRR (intensity)
30
25
20
15
Training
n=117
Race
n=13

2.4 Supplementary Figure 4. Individual Bland & Altman plots

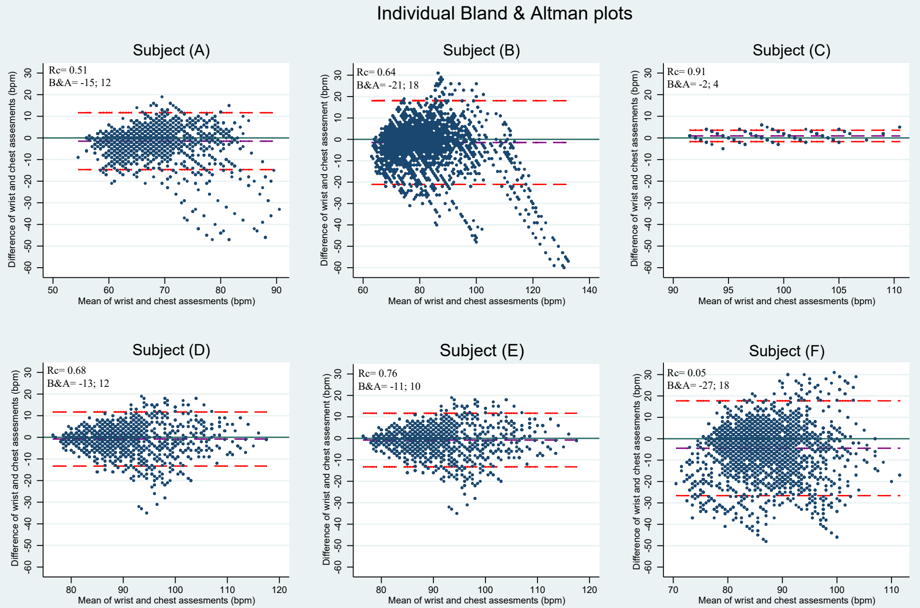

y=0 is line (green) of perfect average agreement

Rc = Lin's Concordance Correlation Coefficient

2.5 Supplementary Table 1 Intensity levels estimated for each participant

Age categories*	RHR	HR_max	HRR	1%	29%	30%	39%	40%	59%	60%	84%	85%	100%
18-30	72	184	112	73	104	106	116	117	138	139	166	167	184
18-30	77	183	106	78	108	109	118	120	140	141	166	167	183
18-30	78	182	104	79	108	109	119	120	139	141	166	167	182
18-30	80	182	102	81	109	110	120	121	140	141	165	166	182
18-30	70	181	111	71	102	103	113	114	136	137	163	164	181
18-30	62	179	117	63	96	97	108	109	131	132	161	161	179
18-30	89	179	90	90	115	116	124	125	142	143	164	165	179
18-30	76	178	102	77	106	107	116	117	136	137	162	163	178
30-45	68	178	110	69	100	101	111	112	133	134	160	161	178
30-45	59	177	107	71	101	102	112	113	133	134	160	161	177
30-45	74	177	103	75	104	105	114	115	135	136	160	161	177
30-45	65	174	109	66	97	98	108	108	130	131	157	158	174
30-45	71	174	103	72	101	102	111	112	132	133	157	158	174
30-45	75	173	98	76	103	104	113	114	133	134	157	158	173
30-45	57	171	114	58	90	91	101	102	124	125	153	154	171
45-70	65	167	102	66	95	96	105	106	125	126	151	152	167
45-70	48	167	119	49	83	84	94	95	118	119	148	149	167
45-70	70	163	104	71	97	98	106	107	125	126	148	149	163
45-70	60	158	98	61	88	89	98	99	118	119	142	143	158
	Fernhall et al. [42]			Very light <29%		Light 30-39%		Moderate 40-59%		Vigorous 60–84%		Very hard >85%	

*to preserve anonymity

Intensity classification (according to Table 1, [21, 45])

2.6 Supplementary Table 2 Subsample and remaining sample characteristics (n=18)

Characteristics	Remaning sample (n=13)	Subsample for agreement evaluation (n=5)
Age	36.9 ± 14.3	31.6 ± 6.0
Sex (male), n %	6 (46.2)	5 (100)
**Disability, n %**		
CP	9 (69.2)	4 (80)
Other	3 (23.1)	1 (20)
(Of those with CP) GMFCS:		
III	2 (22.2)	n/a
IV-V	7 (77.8)	4 (100)
**Medication with potential side-effects on HR, n %**		
Yes	7 (53.8)	1 (20.0)
No	6 (46.2)	4 (80.0)
**Body composition***	*n= 12*	*n=4*
Height (cm)	158.5 ± 10.5	159.5 ± 12.6
Weight (kg)	61.6 ± 17.9	47.4 ± 6.3
BMI (kg/m^2)	24.1 ± 4.4	19.0 ± 4.3
**Heart rate data from Vivosmart 4**		
[a]RHR (bpm)	70.0 ± 9.0	69.8 ± 12.9
HRR (bpm)	105.2 ± 6.0	107.6 ± 10.7
[b]HRmax (bpm)	173.8 ± 8.1	176.8 ± 3.3
**Team Twin sessions & context**		
Training participation	7 ± 4	9 ± 4
Race participation	8 ± 5	8 ± 5
Mean HR during training/race (bpm)	86.4 ± 17.1	96.8 ± 13.8
Mean %HRR during training/race	18.0 ± 13.1	23.7 ± 12.4

Data are presented as mean ±SD, unless stated otherwise.
***n=16 due to missing data**
[a]Mean from the first 7-day period after the two-week calibration.
[b]Estimated from Fernhall et al (42)
Abbreviation; CP = Cerebral Palsy, GMFCS = Gross Motor Function Classification System, BMI = Body Mass Index, HR = Heart rate, RHR = Resting heart rate, HRR = Heart rate reserve, HRmax = Heart rate max, %HRR = percent of heart rate reserve (intensity), bpm = beats per minute

2.7 Supplementary Table 3 Cross tabulation of intensity levels measured by the Vivosmart 4 (Wrist) and the HRM-DUAL (Chest) monitors (n= 41.185). Values are time in HH:MM:SS

Vivosmart 4 (Wrist)	*HRM-DUAL (Chest)*				
	Very light	*light*	*Moderate*	*Vigorous*	Total
Very light	8:51:52	0:03:48	0:24:31	0:00:31	9:20:42
light	0:00:46	1:05:27	0:05:53	0:00:09	1:12:15
Moderate	0:09:16	0:11:36	0:31:22	0:00:58	0:53:12
Vigorous	n/a	0:00:09	n/a	0:00:07	0:00:16
Total	9:01:54	1:21:00	1:01:46	0:01:45	11:26:25

Agreement; 91.6%
Cohen's kappa coefficient; 0.75 (Substantial), estimated by a kappa calculation.
n/a = Not applicable.

2.8 Supplementary Table 4 Illustration of how different HRmax estimations (equations) influence the time spent at each intensity level.

**Equations for HR_{max}**	**Fernhall et al. [42] (Ref.) (210 - 0,56 * age -15,5)**	**Gellish et al [65] (207-(0.7*age))**	**Fox et al. [66] (220-age)**
**Time in zones (%)**			
Very light	79.85	83.05	83,66
Light	10.40	9.62	9,61
Moderate	8.92	6.99	6,48
Vigorous	0.77	0.33	0,25
Very hard	0.01	0.00	0.00

Intensity zone division [21, 45]

2.9 Supplementary File 1 HR transformation example

Data structure for Vivosmart 4

Date and time (DD/MM/YY) (HH:MM:SS)	Time (start-end)	Time spent in each frequency	hear rate (bpm)
19-09-21 11:16:51	00:00:01	00:00:01	104
19-09-21 11:16:52	00:00:04	00:00:03	104
19-09-21 11:16:55	00:00:06	00:00:02	107
19-09-21 11:16:57	00:00:07	00:00:01	111
19-09-21 11:16:58	00:00:10	00:00:03	114
19-09-21 11:17:01	00:00:19	00:00:09	117
19-09-21 11:17:10	00:00:46	00:00:27	112
19-09-21 11:17:37	00:00:53	00:00:07	109
19-09-21 11:17:44	00:00:56	00:00:03	106
19-09-21 11:17:47	00:01:01	00:00:05	103
19-09-21 11:17:52	00:01:12	00:00:11	100
19-09-21 11:18:03	00:01:23	00:00:11	103
19-09-21 11:18:14	00:01:25	00:00:02	107
19-09-21 11:18:16	00:01:31	00:00:06	110
19-09-21 11:18:22	00:01:34	00:00:03	107
19-09-21 11:18:25	00:01:36	00:00:02	103
19-09-21 11:18:27	00:01:38	00:00:02	99
19-09-21 11:18:29	00:01:41	00:00:03	96
19-09-21 11:18:32	00:01:45	00:00:04	93
19-09-21 11:18:36	00:01:51	00:00:06	89
19-09-21 11:18:42	00:01:55	00:00:04	92
19-09-21 11:18:46	00:02:07	00:00:12	96
19-09-21 11:18:58	00:02:12	00:00:05	92
19-09-21 11:19:03	00:02:14	00:00:02	95
19-09-21 11:19:05	00:02:20	00:00:06	99
19-09-21 11:19:11	00:02:22	00:00:02	104
19-09-21 11:19:13	00:02:24	00:00:02	109

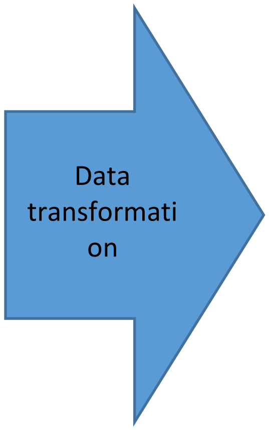

Data structure after transformation

Date + time	Time in sec	heart rate (bpm)
19-09-21 11:16:51	00:00:01	104
19-09-21 11:16:52	00:00:02	104
19-09-21 11:16:53	00:00:03	104
19-09-21 11:16:54	00:00:04	104
19-09-21 11:16:55	00:00:05	107
19-09-21 11:16:56	00:00:06	107
19-09-21 11:16:57	00:00:07	111
19-09-21 11:16:58	00:00:08	114
19-09-21 11:16:59	00:00:09	114
19-09-21 11:17:00	00:00:10	114
19-09-21 11:17:01	00:00:11	117
19-09-21 11:17:02	00:00:12	117
19-09-21 11:17:03	00:00:13	117
19-09-21 11:17:04	00:00:14	117
19-09-21 11:17:05	00:00:15	117
19-09-21 11:17:06	00:00:16	117
19-09-21 11:17:07	00:00:17	117
19-09-21 11:17:08	00:00:18	117
19-09-21 11:17:09	00:00:19	117
19-09-21 11:17:10	00:00:20	112
19-09-21 11:17:11	00:00:21	112
19-09-21 11:17:12	00:00:22	112
19-09-21 11:17:13	00:00:23	112
19-09-21 11:17:14	00:00:24	112
19-09-21 11:17:15	00:00:25	112
19-09-21 11:17:16	00:00:26	112
19-09-21 11:17:17	00:00:27	112

19-09-21 11:19:15	00:02:31	00:00:07	112
19-09-21 11:19:22	00:02:33	00:00:02	108
19-09-21 11:19:24	00:02:34	00:00:01	105

19-09-21 11:17:18	00:00:28	112
19-09-21 11:17:19	00:00:29	112
19-09-21 11:17:20	00:00:30	112
19-09-21 11:17:21	00:00:31	112
19-09-21 11:17:22	00:00:32	112
19-09-21 11:17:23	00:00:33	112
19-09-21 11:17:24	00:00:34	112
19-09-21 11:17:25	00:00:35	112
19-09-21 11:17:26	00:00:36	112
19-09-21 11:17:27	00:00:37	112
19-09-21 11:17:28	00:00:38	112
19-09-21 11:17:29	00:00:39	112
19-09-21 11:17:30	00:00:40	112
19-09-21 11:17:31	00:00:41	112
19-09-21 11:17:32	00:00:42	112
19-09-21 11:17:33	00:00:43	112
19-09-21 11:17:34	00:00:44	112
19-09-21 11:17:35	00:00:45	112
19-09-21 11:17:36	00:00:46	112
19-09-21 11:17:37	00:00:47	109
19-09-21 11:17:38	00:00:48	109
19-09-21 11:17:39	00:00:49	109
19-09-21 11:17:40	00:00:50	109
19-09-21 11:17:41	00:00:51	109
19-09-21 11:17:42	00:00:52	109
19-09-21 11:17:43	00:00:53	109
19-09-21 11:17:44	00:00:54	106
19-09-21 11:17:45	00:00:55	106
19-09-21 11:17:46	00:00:56	106
19-09-21 11:17:47	00:00:57	103
19-09-21 11:17:48	00:00:58	103
19-09-21 11:17:49	00:00:59	103
19-09-21 11:17:50	00:01:00	103

19-09-21 11:17:51	00:01:01	103
19-09-21 11:17:52	00:01:02	100
19-09-21 11:17:53	00:01:03	100
19-09-21 11:17:54	00:01:04	100
19-09-21 11:17:55	00:01:05	100
19-09-21 11:17:56	00:01:06	100
19-09-21 11:17:57	00:01:07	100
19-09-21 11:17:58	00:01:08	100
19-09-21 11:17:59	00:01:09	100
19-09-21 11:18:00	00:01:10	100
19-09-21 11:18:01	00:01:11	100
19-09-21 11:18:02	00:01:12	100
19-09-21 11:18:03	00:01:13	103
19-09-21 11:18:04	00:01:14	103
19-09-21 11:18:05	00:01:15	103
19-09-21 11:18:06	00:01:16	103
19-09-21 11:18:07	00:01:17	103
19-09-21 11:18:08	00:01:18	103
19-09-21 11:18:09	00:01:19	103
19-09-21 11:18:10	00:01:20	103
19-09-21 11:18:11	00:01:21	103
19-09-21 11:18:12	00:01:22	103
19-09-21 11:18:13	00:01:23	103
19-09-21 11:18:14	00:01:24	107
19-09-21 11:18:15	00:01:25	107
19-09-21 11:18:16	00:01:26	110
19-09-21 11:18:17	00:01:27	110
19-09-21 11:18:18	00:01:28	110
19-09-21 11:18:19	00:01:29	110
19-09-21 11:18:20	00:01:30	110
19-09-21 11:18:21	00:01:31	110
19-09-21 11:18:22	00:01:32	107
19-09-21 11:18:23	00:01:33	107

19-09-21 11:18:24	00:01:34	107
19-09-21 11:18:25	00:01:35	103
19-09-21 11:18:26	00:01:36	103
19-09-21 11:18:27	00:01:37	99
19-09-21 11:18:28	00:01:38	99
19-09-21 11:18:29	00:01:39	96
19-09-21 11:18:30	00:01:40	96
19-09-21 11:18:30	00:01:41	96
19-09-21 11:18:31	00:01:42	93
19-09-21 11:18:32	00:01:43	93
19-09-21 11:18:33	00:01:44	93
19-09-21 11:18:34	00:01:45	93
19-09-21 11:18:35	00:01:46	89
19-09-21 11:18:36	00:01:47	89
19-09-21 11:18:37	00:01:48	89
19-09-21 11:18:38	00:01:49	89
19-09-21 11:18:39	00:01:50	89
19-09-21 11:18:40	00:01:51	89
19-09-21 11:18:41	00:01:52	92
19-09-21 11:18:42	00:01:53	92
19-09-21 11:18:43	00:01:54	92
19-09-21 11:18:44	00:01:55	92
19-09-21 11:18:45	00:01:56	96
19-09-21 11:18:46	00:01:57	96
19-09-21 11:18:47	00:01:58	96
19-09-21 11:18:48	00:01:59	96
19-09-21 11:18:49	00:02:00	96
19-09-21 11:18:50	00:02:01	96
19-09-21 11:18:51	00:02:02	96
19-09-21 11:18:52	00:02:03	96
19-09-21 11:18:53	00:02:04	96
19-09-21 11:18:54	00:02:05	96
19-09-21 11:18:55	00:02:06	96

19-09-21 11:18:56	00:02:07	96
19-09-21 11:18:57	00:02:08	92
19-09-21 11:18:58	00:02:09	92
19-09-21 11:18:59	00:02:10	92
19-09-21 11:19:00	00:02:11	92
19-09-21 11:19:01	00:02:12	92
19-09-21 11:19:02	00:02:13	95
19-09-21 11:19:03	00:02:14	95
19-09-21 11:19:04	00:02:15	99
19-09-21 11:19:05	00:02:16	99
19-09-21 11:19:06	00:02:17	99
19-09-21 11:19:07	00:02:18	99
19-09-21 11:19:08	00:02:19	99
19-09-21 11:19:09	00:02:20	99
19-09-21 11:19:10	00:02:21	104
19-09-21 11:19:11	00:02:22	104
19-09-21 11:19:12	00:02:23	109
19-09-21 11:19:13	00:02:24	109
19-09-21 11:19:14	00:02:25	112
19-09-21 11:19:15	00:02:26	112
19-09-21 11:19:16	00:02:27	112
19-09-21 11:19:17	00:02:28	112
19-09-21 11:19:18	00:02:29	112
19-09-21 11:19:19	00:02:30	112
19-09-21 11:19:20	00:02:31	112
19-09-21 11:19:21	00:02:32	108
19-09-21 11:19:22	00:02:33	108
19-09-21 11:19:23	00:02:34	105
